# PREFRONTAL BLOOD FLOW DURING DUAL-TASK WALKING IN ADULTS WITH AND WITHOUT MILD COGNITIVE IMPAIRMENT

**DOI:** 10.1093/geroni/igac059.2267

**Published:** 2022-12-20

**Authors:** Cristina Udina Argilaga, Stella Avtzi, Turgut Durduran, Andrea Rosso, Miriam Mota, Joan Ars, Marco Inzitari

**Affiliations:** Parc Sanitari Pere Virgili, Barcelona, Catalonia, Spain; Institut de Ciències Fotòniques, The Barcelona Institute of Science and Technology, Barcelona, Catalonia, Spain; Institut de Ciències Fotòniques, The Barcelona Institute of Science and Technology, Barcelona, Catalonia, Spain; University of Pittsburgh, Pittsburgh, Pennsylvania, United States; Vall Hebron Research Institute (VHIR), Barcelona, Catalonia, Spain; Parc Sanitari Pere Virgili, Barcelona, Catalonia, Spain; Parc Sanitari Pere Virgili, Barcelona, Catalonia, Spain

## Abstract

**Background:**

Blood flow differences in the prefrontal cortex (PFC) during dual-task walking are thought to indicate various degrees of neural efficiency. Individuals with poorer neural resources might need higher activation to meet behavioral performance. We aim to assess PFC cerebral blood flow (CBF) among older adults with and without mild cognitive impairment (MCI) during dual-task using functional Diffuse Correlation Spectroscopy (fDCS).

**Methods:**

We assessed PFC CBF with DCS during dual-task paradigm: 1)Normal Walk(NW); 2)Forward-count(FWC); 3)Backward-count(BWC); 4)Obstacle negotiation(WWO). We assessed demographics, clinical variables, physical and cognitive function in those with MCI vs normal cognition (NC). Linear mixed effects models assessed changes of CBF across the tests in the dual-task paradigm and differences between MCI and NC.

**Results:**

49 older adults (median age=78 years, 51% women, 34 MCI) were included. MCI were older, with higher frailty, polypharmacy and comorbidity. Compared to NC, MCI showed worse cognitive and physical performance scores and lower Gait Speed (GS) during NW and WWO but not during FWC and BWC. N=12 were unable to perform BWC. CBF change from NW to FWC was higher in MCI compared to NC (estimate=0.35, 95%CI [0.03, 0.67], p=0.03). CBF change from NW to BWC and WWO was not different between groups. There was no effect of age or clinical covariates.

**Conclusions:**

Higher NW-FWC CBF change seems due to the cognitive load of FWC in MCI. Higher activation in MCI compared to healthier counterparts could be explained by compensatory mechanisms. Further research should focus on better understanding dual-task related neural mechanisms.

